# The association of adelmidrol with sodium hyaluronate displays beneficial properties against bladder changes following spinal cord injury in mice

**DOI:** 10.1371/journal.pone.0208730

**Published:** 2019-01-17

**Authors:** Michela Campolo, Rosalba Siracusa, Marika Cordaro, Alessia Filippone, Enrico Gugliandolo, Alessio F. Peritore, Daniela Impellizzeri, Rosalia Crupi, Irene Paterniti, Salvatore Cuzzocrea

**Affiliations:** 1 University of Messina, Department of Chemical, Biological, Pharmaceutical and Environmental Sciences, Messina, Italy; 2 Saint Louis University School of Medicine, Department of Pharmacological and Physiological Science, Saint Louis, United States of America; University Medical Center Utrecht, NETHERLANDS

## Abstract

The disruption of coordinated control between the brain, spinal cord and peripheral nervous system caused by spinal cord injury (SCI) leads to several secondary pathological conditions, including lower urinary tract dysfunction. In fact, urinary tract dysfunction associated with SCI is urinary dysfunction could be a consequence of a lack of neuroregeneration of supraspinal pathways that control bladder function. The object of the current research was to explore the effects of adelmidrol + sodium hyaluronate, on bladder damage generated after SCI in mice. Spinal cord was exposed via laminectomy, and SCI was induced by extradural compression at T6 to T7 level, by an aneurysm clip with a closing force of 24 g. Mice were treated intravesically with adelmidrol + sodium hyaluronate daily for 48 h and 7 days after SCI. Adelmidrol + sodium hyaluronate reduced significantly mast cell degranulation and down-regulated the nuclear factor-κB pathway in the bladder after SCI both at 48 h and 7days. Moreover, adelmidrol + sodium hyaluronate reduced nerve growth factor expression, suggesting an association between neurotrophins and bladder pressure. At 7 days after SCI, the bladder was characterized by a marked bacterial infection and proteinuria; surprisingly, adelmidrol + sodium hyaluronate reduced significantly both parameters. These data show the protective roles of adelmidrol + sodium hyaluronate on bladder following SCI, highlighting a potential therapeutic target for the reduction of bladder changes.

## Introduction

Spinal cord injury (SCI) typically causes permanent impairment of motor and sensory functions, which outcome in a huge socioeconomic burden [1–3]. The pathogenesis of SCI starts from primary mechanical damage to the spinal cord followed by secondary injury. The secondary injury phase in SCI, caused by oxidative stress, edema, inflammatory reactions and other processes, has been proven to function as a significant therapeutic window [4–6]. The disruption of coordinated control caused by SCI, between central and peripheral nervous system, leads to several secondary pathological conditions including alteration of bladder function [7]. Specifically, the phenomena responsible for the development of bladder damage regards the C-fiber urinary bladder afferent that represents the prevailing afferent route transporting impulses from the spinal tract to control the micturition reflex [8, 9]; in particular the sensitization of C-fiber urinary bladder afferents to various stimuli because their considerable plasticity and a local effector function of afferent C-fiber endings, contributes to neurogenic inflammation [10]. Adelmidrol, a derivative of azelaic acid, is an analogue of palmitoylethanolamide (PEA), an endogenous fatty acid amide belonging to the family of the N-acylethanolamines. Recent evidence indicates that adelmidrol effects may be due to the control of mast cell (MC) degranulation [11]. The latter appear to be involved in the pathogenesis of interstitial cystitis [12]. In fact, topical adelmidrol treatment improves MC granule density, proposing a decrease in their degranulation [13, 14]. In addition, this molecule exhibited favorable effects in a pilot study on mild atopic dermatitis [15] as well as beneficial properties on acute and chronic inflammation [16]. Based on these findings, we focused our attention on a new formulation of adelmidrol + sodium hyaluronate. This compound is a treatment able to optimize the restoring process of the urothelium coating integrity altered by dysmetabolic conditions associated with inflammatory events of different origins; this process is favored by the intravesical installation of hyaluronic acid that forms an impermeable barrier in the bladder. Moreover, our hypothesis was supported by a recent study highlighting the anti-inflammatory effect of this association on in vivo model of interstitial cystitis/painful bladder syndrome (IC/PBS) and in IC/BPS patients [17, 18]; as well as positive effects in a rat model of osteoarthritis [19]. The intentions of this study were to evaluate the acute and chronic effects of intravesical adelmidrol + sodium hyaluronate injection as a new therapeutic approach to treat bladder damage induced by SCI.

## Materials and methods

### Materials

2% adelmidrol + 0.1% sodium hyaluronate was obtained from Epitech group S.p.A (Saccolongo, Italy). All other chemicals were obtained from commercial founts and were of the highest grade available. All stock solutions were prepared in non-pyrogenic saline (0.9% NaCl, Baxter, Milan, Italy).

### Animals

Male CD1 mice at 4–5weeks old, (25–30 g; Envigo, Italy) were housed in a controlled environment and provided with standard rodent food and water. Mice were located in stainless steel cages in a room kept at 22 ± 1°C with a 12-h dark, 12-h light cycle. The animals were familiarized to their habitat for 1 week and had access to rodent standard diet and water ad libitum. University of Messina Review Board for the care of animals approved the study. Animal care was in accordance with the novel legislation for the protection of animals used for scientific purposes (D.Lgs 2014/26 and EU Directive 2010/63).

### Surgical procedures for SCI

Animals were anesthetized with intra-peritoneal xylazine and ketamine (0.16 and 2.6 mg/kg body weight, respectively). Spinal cord was uncovered via laminectomy, and SCI was induced by extradural compression for 1 minutes at T6 to T7 level, by an aneurysm clip with a closing force of 24 g. Following surgery, to restore the blood volume lost during surgery, 1.0 ml of saline was given subcutaneously. Throughout surgery and recovery from anesthesia, animals were covered with a heat towel and positioned on a warm heating pad. They were individually housed in a temperature controlled room at 27°C for 24 h. Diet and water were provided ad libitum. During this phase, the animals’ bladders were manually voided daily until the animals were able to recover normal bladder function.

Mice were distributed randomly into several groups (30 mice for each group of which 10 for histological analysis, immunohistochemistry and immunofluorescence staining, 10 for western blot analysis and 10 for microbiological evaluation). The experimenter was unaware to the treatment groups during surgery.

Mice were randomly allocated as follows:

1. Sham+ vehicle group: animals were exposed to the surgical procedures, but the aneurysm clip was not applied and they received saline in bladder.
2. Sham+ 2% adelmidrol and 0.1% sodium hyaluronate: same as the Sham+ vehicle group, but adelmidrol + sodium hyaluronate (100μl/mouse) intravesical instillation was administered at 1, 6 h and 24 h after injury.
3. SCI+ vehicle group: animals were subjected to SCI and intravesical administration of vehicle (saline) 1, 6 and 24 h after injury.
4. SCI+2% adelmidrol and 0.1% sodium hyaluronate: same as the SCI+ vehicle group, but adelmidrol + sodium hyaluronate (100μl/mouse) was administered in bladder via transurethral catheter at 1, 6 h and 24 h after injury.

SCI mice were divided in two separate groups [20]: early phase of recovery [16], 2 days; intermediate phase of recovery at 7 days [21]. Mice in the chronic study groups received adelmidrol + sodium hyaluronate (100 μl/mouse) in bladder via transurethral catheter after 1 and 6 h, and then daily until 7 days.

At the end of each experiment animals were sacrificed by anaesthetic (xylazine and ketamine) overdose, specifically: the groups of the early stage were sacrificed 2 days after SCI; the groups of the intermediate phase of recovery were sacrificed 7 days after SCI. For all groups, bladder and urine were collected. The dose of adelmidrol + sodium hyaluronate was based on our previous study on cystitis [22]. Intravesical administration was performed by urinary bladder catheterization according to a previous report [23].

### Histological analysis

For histopathological studies, 48 h and 7 days following SCI, the animals were sacrificed and biopsies of bladder were fixed in buffered formaldehyde solution (10% in phosphate buffered saline (PBS)) at room temperature, dehydrated by graded ethanol, embedded in Paraplast (Bio-Optica, Milano, Italy) and cut into 7-μm-thick sections. Bladder sections were deparaffinized with xylene, stained with hematoxylin/eosin (H&E, Bio-Optica, Milano, Italy) and studied using Axiovision Zeiss (Milan, Italy) microscope [16]. The stained sections were scored by two researchers in a blind manner, and the degree of inflammation was evaluated according to a score from 0 to 5, as follows: 0 = absence of inflammation, 1 = bland inflammation, 2 = mild/moderate inflammation, 3 = modest inflammation, 4 = modest/severe inflammation and 5 = severe inflammation.

### Periodic Acid Schiff staining (PAS)

PAS staining is routinely used to visualize sugar moieties. It provides insight into general tissue structure, comparable to hematoxylin/eosin staining. Combined, the two stains allow visualization of granular glycogen deposits. Three individuals in a blinded fashion scored the number of stained granules in bladder sections. The score ranged from 0, no staining of glycogen granules to 3, the most intense staining. Previously it was found that an increase in glycogen score coincided with a change in the location of deposition, from only the serosal side at a score of 1 to increasingly throughout the muscle at score 2 and severe staining up to the urothelium at the score of 3 [24].

### Toluidine blue staining

Sections were deparaffinized in xylene and dehydrated by a graded successions of ethanol, 5 minutes in each solution. The sections were next sited in water for 5 minutes, relocated to toluidine blue for 4 minutes and then blotted cautiously. Sections were positioned in absolute alcohol for 1 minute, cleared in xylene, and fixed on glass slides using Eukitt (Bio-Optica, Milan, Italy). The number of metachromatic stained mast cells was obtained by counting five high-power fields (40×) per section using an Axiovision Zeiss (Milan, Italy) microscope.

### Immunofluorescence for zonula occludens-1 (ZO-1) and nerve growth factor (NGF)

After deparaffinization and rehydration, detection of ZO-1 and NGF was carried out after boiling the sections in 0.1 M citrate buffer for 1 minute. Non-specific adsorption was minimalized by incubating in 2% (vol/vol) standard goat serum in PBS for 20 minutes. Bladder sections were incubated overnight with murine monoclonal anti-ZO-1 (1:100, Santa Cruz Biotechnology, Santa Cruz, CA, USA), or mouse monoclonal anti-NGF (1:100, Santa Cruz Biotechnology, CA, USA) antibodies at 37°C in a humidified oxygen and nitrogen chamber. Sections were then incubated with secondary antibody Texas Red-conjugated anti-rabbit Alexa Fluor-594 antibody (1:1000 in PBS, vol/vol Molecular Probes, Monza, Italy) for 1 h at 37°C. Nuclei were stained by adding 2 μg/ml 4′,6′-diamidino-2-phenylindole (DAPI; Hoechst, Frankfurt, Germany) in PBS. Sections were observed at 40× and 100× magnifications by a Leica DM2000 microscope (Leica, Milan, Italy). Optical sections of samples were obtained by a HeNe laser (543 nm), an UV laser (361 to 365 nm) and an argon laser (458 nm) at a 1 minute, 2 seconds scanning rapidity with up to 8 averages; 1.5 μm sections were attained using a pinhole of 250. Examining the most luminously labeled pixels and using settings that allowed clear visualization of structural details, while keeping the maximum pixel intensities close to 200, established contrast and brightness. The same settings were used for all images obtained from the other samples that had been processed in parallel. Digital images were cropped and figure montages ready using Adobe Photoshop 7.0 (Adobe Systems; Palo Alto, California, United States).

### Western blot analysis

Nuclear and cytosolic extracts were prepared as described previously [25]. Bladder tissue from each animals were suspended in extraction buffer A containing PMSF 0.2 mM, pepstatin A 0.15 mM, leupeptin 20 mM, sodium orthovanadate 1 mM, homogenized for 2 minutes, and centrifuged for 4 minutes at 4°C at 12,000 rpm. Supernatants represented the cytosolic fraction. The pellets, containing enriched nuclei, were resuspended in buffer B containing 1% Triton X-100, NaCl 150 mM, Tris–HCl pH 7.4 10 mM, EGTA 1 mM, EDTA 1 mM, PMSF 0.2 mM, leupeptin 20 mM, and sodium orthovanadate 0.2 mM. After centrifugation 10 minutes at 12,000 rpm at 4°C, the supernatants containing the nuclear protein were stored at **−**80°C for further analysis. The levels of IκB-α and inducible nitric oxide synthase (iNOS) were measured in cytosolic fractions. NF-κB p65 was measured in nuclear fractions from bladder collected 48 hours after SCI. The filters were blocked with 1x PBS and 5% (w/v) non-fat desiccated milk for 40 minutes at room temperature and successively probed with one of the following primary antibodies: mouse monoclonal anti-NF-κB p65 (1:500; Santa Cruz Biotechnology, CA, USA), mouse monoclonal anti-IκBα (1:500; Santa Cruz Biotechnology, CA, USA), and rabbit polyclonal anti-iNOS (1:500, Cayman, Vinci-Biochem, Italy) at 4°C overnight in 1× PBS, 5% (w/v), nonfat dried milk, and 0.1% Tween-20. Membranes were incubated with peroxidase conjugated goat anti-rabbit IgG or peroxidase conjugated bovine anti-mouse IgG secondary antibody (1:2000, Jackson ImmunoResearch, West Grove, PA, USA) for 1 hour at room temperature. To assess that blots were loaded with equal volumes of protein lysates, they were probed with either a mouse monoclonal β-actin antibody (1:5000; Santa Cruz Biotechnology, CA, USA) for cytosolic proteins or a mouse monoclonal lamin A/C antibody (1:5000; Santa Cruz Biotechnology, CA, USA) for nuclear proteins. Signals were detected with Super Signal West Pico Chemiluminescent Substrate according to the producer's instructions (Pierce Thermo Scientific, Rockford, IL, USA). The relative expression of protein bands was quantified by densitometric scanning of the X-ray films utilizing a GS-700 Imaging Densitometer (GS-700, Bio-Rad Laboratories, Milan, Italy) and a computer program (Image J), and standardized to β-actin and Lamin A/C levels.

### Immunostaining of chymase and tryptase

Briefly, At 48 h and 7 days following SCI, the bladder tissues were fixed in 10% buffered formaldehyde and 7 μm sections were prepared from paraffin embedded tissues. After deparaffinization, endogenous peroxidase was quenched with 0.3% H_2_O_2_ in 60% methanol for 30 minutes. The sections were permeabilized with 0.1% Triton X-100 in PBS for 20 minutes. Non-specific adsorption was minimized by incubating the section in 2% normal goat serum in PBS for 20 minutes. Endogenous biotin or avidin binding sites were blocked by sequential incubation for 15 minutes with avidin and biotin [26]. Slides were incubated overnight with either an anti-chymase mouse monoclonal antibody (Santa Cruz Biotechnology, CA, USA, 1:250 in PBS, v/v) or an anti-tryptase mouse monoclonal antibody (Santa Cruz Biotechnology, CA, USA, 1:250 in PBS, v/v). Slides were then washed with PBS and incubated with secondary antibody. Specific labeling was identified with avidin-biotin peroxidase complex and a biotin conjugated goat anti-rabbit IgG (Vector Labs Inc., Burlingame, CA). To verify antibody-binding specificity, some slides were also incubated with only primary antibody or secondary antibody; no positive staining was found. Immunohistochemical images were evaluated by densitometric analysis using an imaging densitometer (AxioVision, Zeiss, Milan, Italy).

### Proteinuria assay

Urine was collected 7 days after SCI (day of sacrifice) in sterile tubes by firm palpation of the bladder and stored at -20°C. Urine samples were tested for protein to assess proteinuria using the Bradford assay (Bio-Rad Laboratories, Inc., Hercules, CA) [27].

### Microbiological evaluation

At 7 days post-SCI, the bacterial burdens of bladder and urine were evaluated by plating serial dilutions of organ homogenates and urine onto Lysogeny broth (LB) agar (Sigma- Aldrich), a nutritionally rich medium and counting the colony-forming units to evaluate bacteria proliferation.

### Statistical evaluation

All values in the figures and text are expressed as mean ± standard deviation (SD), of N observations. For *in vivo* studies N represents the number of animals studied. Results displayed in the figures are representative of at minimum 3 experiments performed on diverse *in vivo* experimental days. The results were examined by one-way analysis of variance followed by a Bonferroni post-hoc test for multiple comparisons. A p-value of less than 0.05 was considered significant.

## Results

### Adelmidrol + sodium hyaluronate acute effects on bladder histological injury after SCI

**Fig 1:**
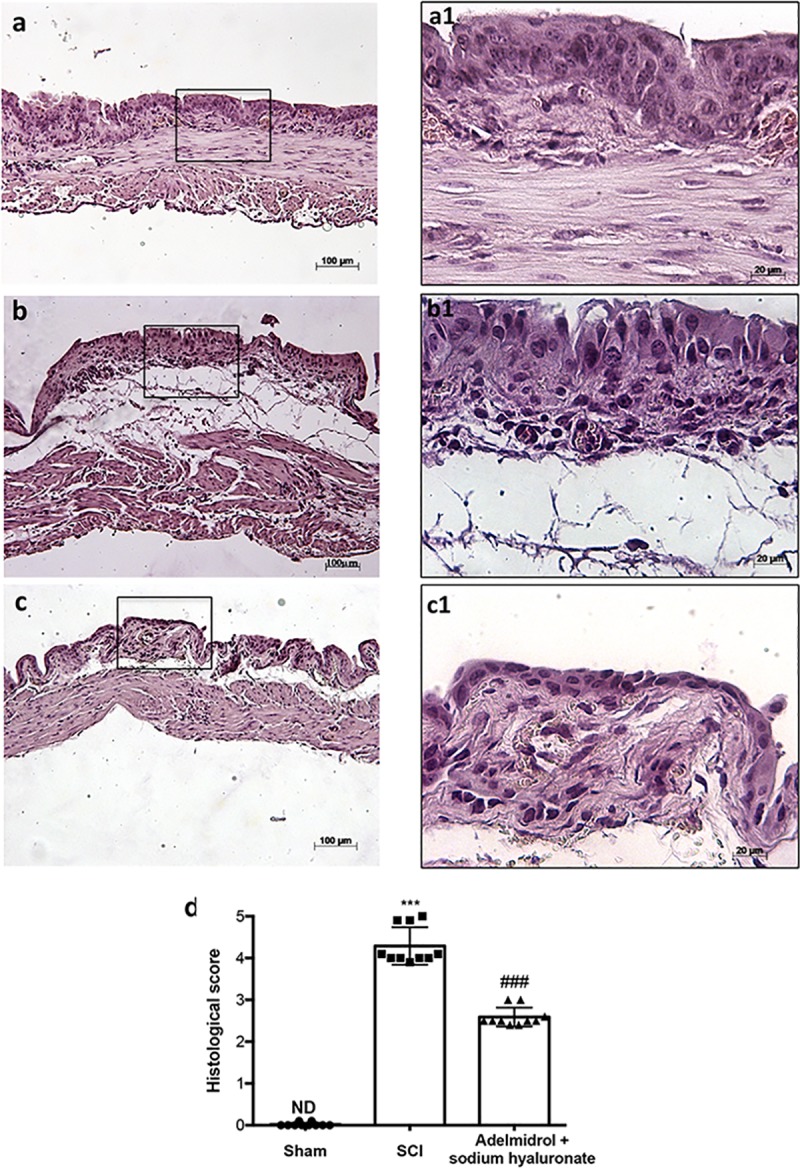
Acute effect of adelmidrol + sodium hyaluronate on histological parameters after SCI. Control mice showed a normal musculature and no signs of edema (a, a1), whereas at 48 h post-SCI, mice showed clear signs of sub-mucosal edema and epithelial ulceration (b, b1). Adelmidrol + sodium hyaluronate treatment restored bladder structure (c, c1). The histological score summarizes these data (d) ***p< 0.001 *vs* Sham; ^###^p< 0.001 *vs* SCI.

Bladder histological examination showed significant alterations 48 h post-SCI, with severe signs of sub-mucosal edema and epithelial ulceration (Fig 1B; higher magnification in 1B1, and relative histological analysis in 1D), compared to control mice, where an intact urothelium and normal musculature were evident with no signs of edema (Fig 1A; higher magnification in 1A1, and relative histological analysis in 1D). Adelmidrol + sodium hyaluronate treatment significantly reduced histological damage (Fig 1C; higher magnification in 1C1, and relative histological analysis in 1D).

**Fig 2:**
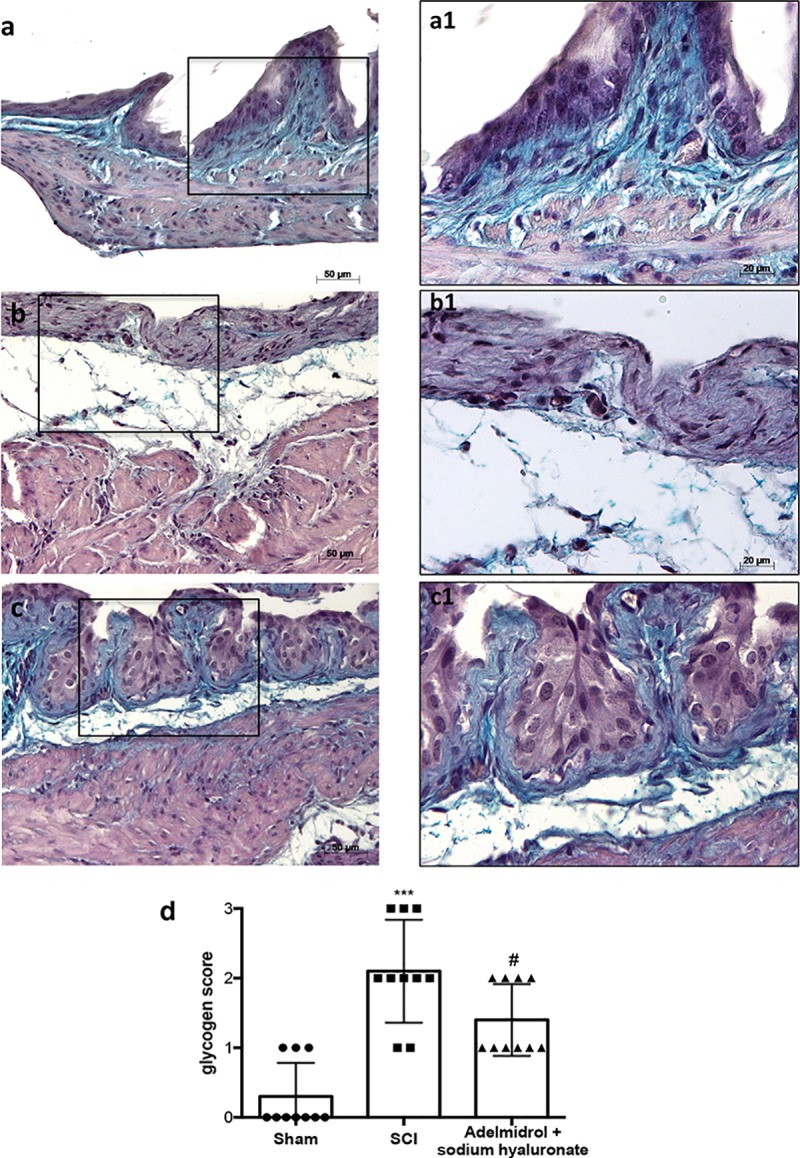
Acute effect of adelmidrol + sodium hyaluronate on mucus-producing cells after SCI. PAS staining identifies granular glycogen deposits in bladder tissue and was performed 48 h after SCI. Control mice showed no staining of glycogen granules (a, a1). Mice subjected to SCI presented an augmented staining of glycogen in the bladder (b, b1), while mice treated with adelmidrol + sodium hyaluronate showed decreased staining (c, c1). These data are also visible in the glycogen score (d) ***p< 0.001 *vs* Sham; ^#^p< 0.05 *vs* SCI.

In order to investigate the effect of adelmidrol + sodium hyaluronate on mucus‐producing cells, sections were stained with PAS to identify the granular glycogen deposits in bladder tissue (Fig 2; S1 Table). Mice subjected to SCI presented an increased number of positive staining in the bladder (Fig 2B; higher magnification in 2B1, and relative graph in 2D) compared to the sham group where no staining of glycogen granules was found (Fig 2A; higher magnification in 2A1 and relative graph in 2D;). PAS staining was lower in mice treated intravesically with adelmidrol + sodium hyaluronate (Fig 2C; higher magnification in 2C1 and relative graph in 2D).

### Adelmidrol + sodium hyaluronate acute effects in bladder: mast cell degranulation following SCI

**Fig 3:**
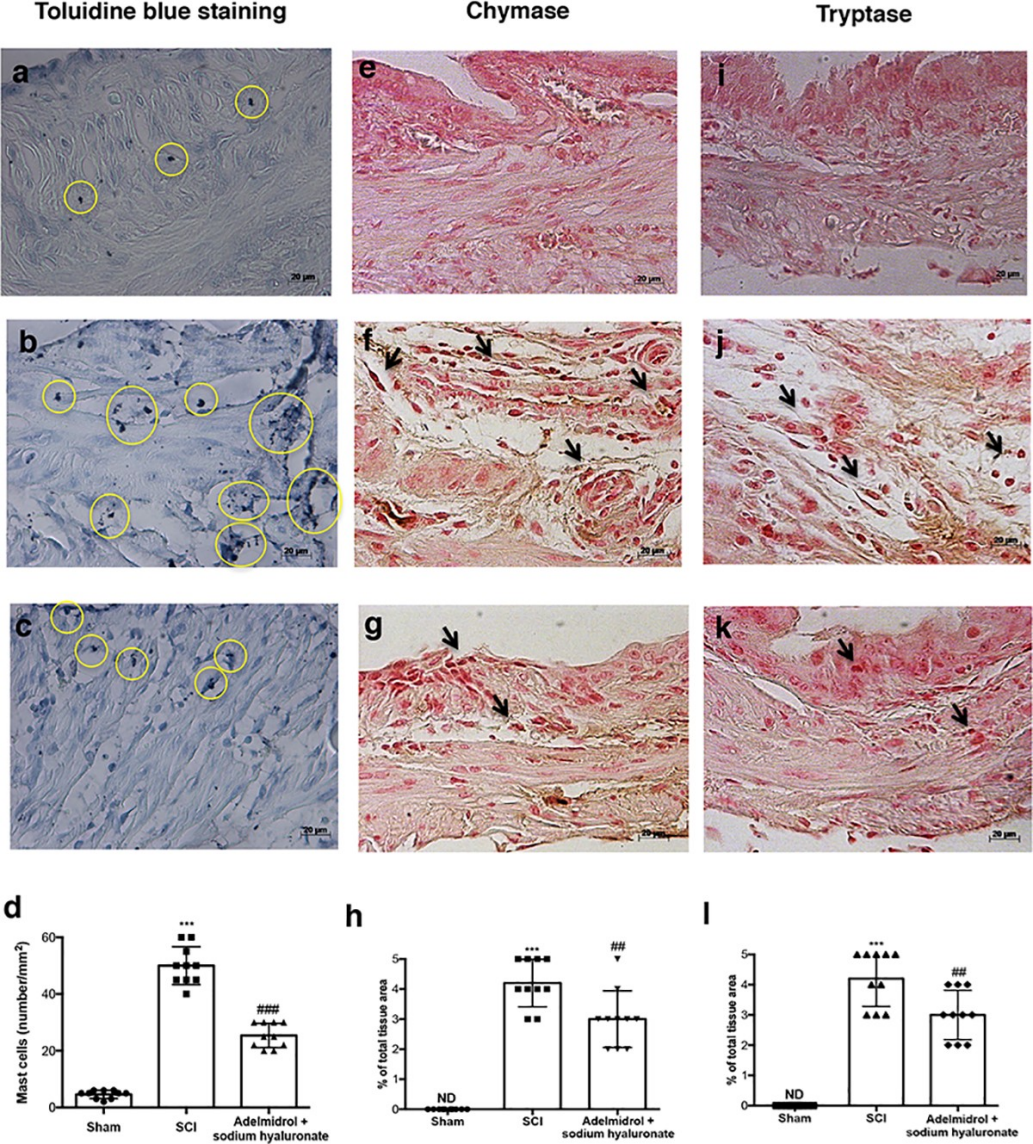
Acute effects of adelmidrol + sodium hyaluronate on MC and on levels of chymase and tryptase. Toluidine blue staining was used to identify mast cell infiltration (encircled), characterized by dark lilac blue granules: (a) Sham group; (b) SCI group; (c) SCI+ 2% adelmidrol and 0.1% sodium hyaluronate group. (d) Mast cell number per unit area of tissue (mast cell density). Figures are illustrative of at least 3 distinct experiments. Values are means ± SD of 10 animals for each group. ***p<0.001 *vs* Sham, ^###^p<0.01 *vs* SCI. Immunohistochemical analysis showed no staining for chymase and tryptase in the Sham group (e and i). Increased chymase and tryptase expression was observed in bladder collected 48 h after SCI (f and j), while low levels of these proteins were found in mice treated with adelmidrol + sodium hyaluronate (g and k). The data are also presented graphically as percentage of total tissue area (h and l). h) ***p< 0.001 *vs* Sham; ^##^p< 0.01 *vs* SCI; l) ***p< 0.001 *vs* Sham; ^##^p< 0.01 *vs* SCI.

The histological pattern of bladder following SCI appeared to correlate with cellular changes. In particular (Fig 3; S2 Table), the presence of MCs was observed in bladder tissues collected 48 h after SCI (Fig 3B and relative graph in 3D.). MCs were identified as Cells with metachromatic granules stained with toluidine blue. Significantly less MCs density and degranulation were observed in SCI bladder collected from mice treated with adelmidrol + sodium hyaluronate (Fig 3C and relative graph in 3D;). Granules were absent in bladder tissues from sham-operated mice (Fig 3A and relative graph in 3D.)

In order to test whether adelmidrol + sodium hyaluronate may modulate and direct the inflammatory response by regulating levels of mast cell-derived serine peptidases, immunohistochemistry was used to assess the expression of chymase and tryptase. Staining for chymase and tryptase was absent in the bladder from sham-operated mice (Fig 3E and 3I; relative graphs 3H and 3L). A substantial increase in chymase and tryptase-positive staining was found principally localized in MCs in bladder collected 48 h after SCI (Fig 3F and 3J; relative graphs 3H and 3L). Immunoreactivity for chymase and tryptase was significantly attenuated with adelmidrol + sodium hyaluronate treatment (Fig 3G and 3K; relative graphs 3H and 3L).

### Adelmidrol + sodium hyaluronate acute effects on bladder tight junctions and on urinary NGF after SCI

**Fig 4:**
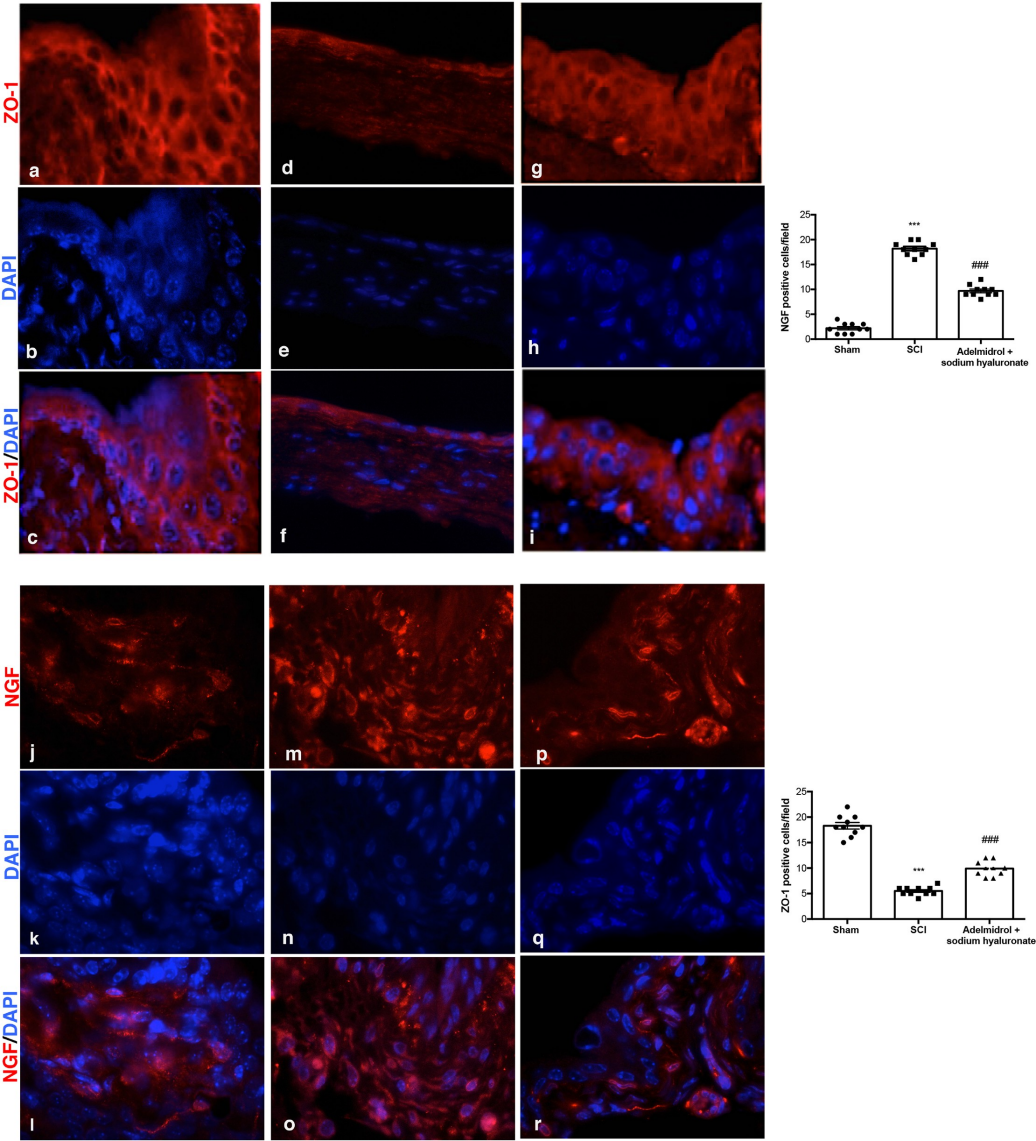
Acute effects of adelmidrol + sodium hyaluronate on ZO-1 and NGF expression in bladder after SCI. Immunofluorescence for ZO-1 expression (red) in sham mice (a-c), SCI group (d-f) and SCI mice treated with adelmidrol + sodium hyaluronate (g-i). Immunofluorescence for NGF expression (red) in sham group (j-l), SCI group (m-o) and SCI mice treated with adelmidrol + sodium hyaluronate (p-r). Data are demonstrative of at least three separate experiments. Images are representative of all animals in each group. The graphs next to the respective panel represent the positive ZO-1 and NGF cells. All images were digitalized at a resolution of 8 bits into an array of 2048 x 2048 pixels. Pictures were captured at 100x magnification.

Acute inflammation leads to endothelial tight junction disassembly (ZO-1;Fig 4; S3 Table); accordingly, immunofluorescence staining evidenced a decreased expression of ZO-1 in bladders of mice of SCI group (Fig 4D–4F, see relative graph;) compared to control animals (Fig 4A–4C, see relative graph;). Adelmidrol + sodium hyaluronate treatment increased significantly ZO-1 expression (Fig 4G–4I, see relative graph).

It is known that the urinary levels of NGF are increased in patients with bladder dysfunction of non-neurogenic origin; therefore, we researched the expression of NGF by immunofluorescence staining (Fig 4; S4 Table). An increase in NGF positive staining was discovered in the bladder of mice subjected to SCI (Fig 4M–4O, see relative graph;) compared to Sham group (Fig 4J–4L, see relative graph;); while adelmidrol + sodium hyaluronate treatment was able to reduce NGF positive staining (Fig 4P–4R, see relative graph).

### Adelmidrol + sodium hyaluronate acute effects on inflammation in bladder after SCI

**Fig 5:**
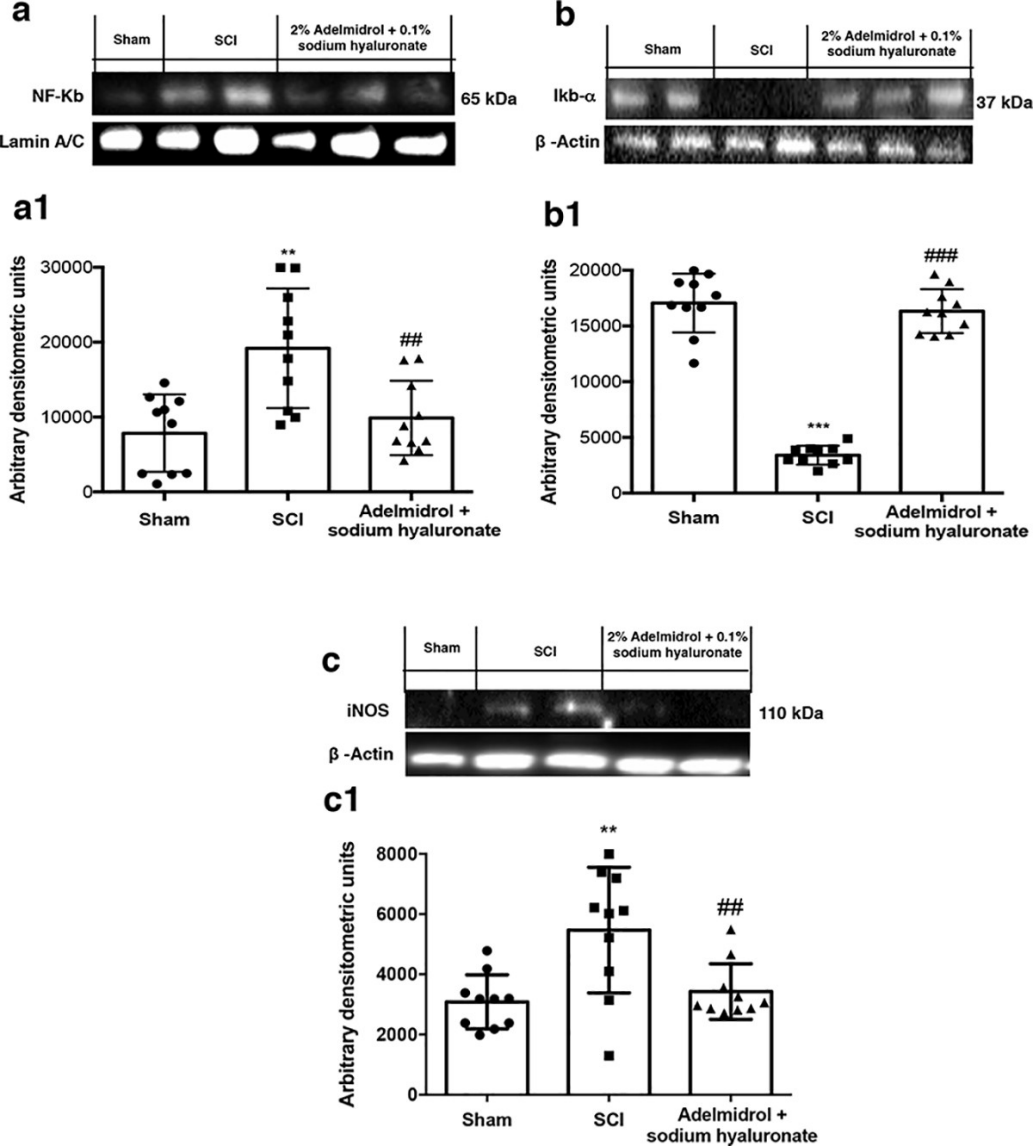
Acute effects of adelmidrol + sodium hyaluronate on NF-κB pathway and iNOS expression after SCI. Representative western blots showing the effects of adelmidrol + sodium hyaluronate on: (a, a1) NF-κB p65 nuclear translocation, (b, b1) IκB-α degradation and (c, c1) iNOS expression at 48 h post-SCI. Adelmidrol + sodium hyaluronate treatment reduced NF-κB p65 translocation (a, a1), IκB-α degradation (b, b1), and iNOS expression (c, c1). Data are illustrative of at minimum three independent experiments. a) **p < 0.01 *vs* Sham; ^##^p < 0.01 *vs* SCI; b) ***p < 0.001 *vs* Sham; ^###^p < 0.001 *vs* SCI; c) **p < 0.01 *vs* Sham; ^##^p < 0.01 *vs* SCI.

To characterize bladder inflammatory state after SCI and the pathway(s) involved, expression of NF-κB, IκB-α and iNOS were evaluated by Western blot (Fig 5, S1 Fig). SCI induced a marked increase in nuclear translocation of NF-κB p65 in bladder compared to sham animals (Fig 5A, densitometric analysis in 5A1; see S1 Fig). Treatment with adelmidrol + sodium hyaluronate significantly prevented NF-κB traslocation (Fig 5A, densitometric analysis in 5A1; see S1 Fig). A basal level of IκB-α was observed in bladder tissues from sham animals whereas cytoplasmic IκB-α degradation was substantially increased in bladder after SCI (Fig 5B, densitometric analysis in 5B1; see S1 Fig). Intravesical administration of adelmidrol + sodium hyaluronate prevented IκB-α degradation (Fig 5B, densitometric analysis in 5B1; see S1 Fig). Moreover, SCI mice showed a significant increase in iNOS expression compared to sham mice (Fig 5C, densitometric analysis in 5C1; see S1 Fig). Adelmidrol + sodium hyaluronate treatment reduced levels of this inflammatory mediator (Fig 5C, densitometric analysis in 5C1; see S1 Fig).

### Chronic effects of adelmidrol + sodium hyaluronate on bladder 7 days after SCI

**Fig 6:**
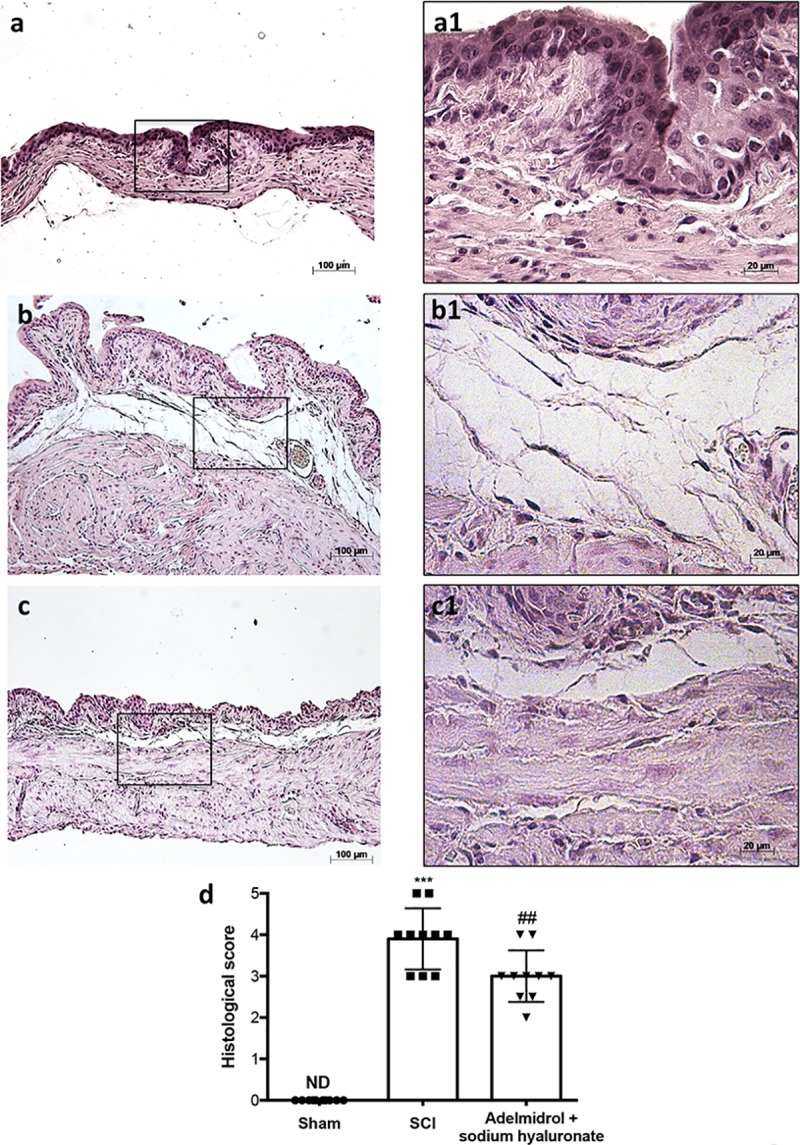
Chronic effects of adelmidrol + sodium hyaluronate on histological alteration after SCI. Control mice showed a normal musculature and no signs of edema (a, a1), whereas at 7 days post-SCI, mice showed evident signs of sub-mucosal edema and epithelial ulceration (b, b1). Adelmidrol + sodium hyaluronate treatment restored bladder structure (c, c1). These data are also visible in the histological score (d) ***p< 0.001 *vs* Sham; ^##^p< 0.01 *vs* SCI.

Mild edema of the submucosa and lamina propria of bladder was evident in mice subjected to SCI (Fig 6B; higher magnification in 6B1 and relative histological analysis in 6D) with neutrophil infiltrates, while no evident sign of damage was observed in the sham group (Fig 6A; higher magnification in a1 and relative histological analysis in 6D). Adelmidrol + sodium hyaluronate treatment significantly reduced bladder tissue damage 7 days after SCI (Fig 6C; higher magnification in 6C1 and relative histological analysis in 6D).

**Fig 7:**
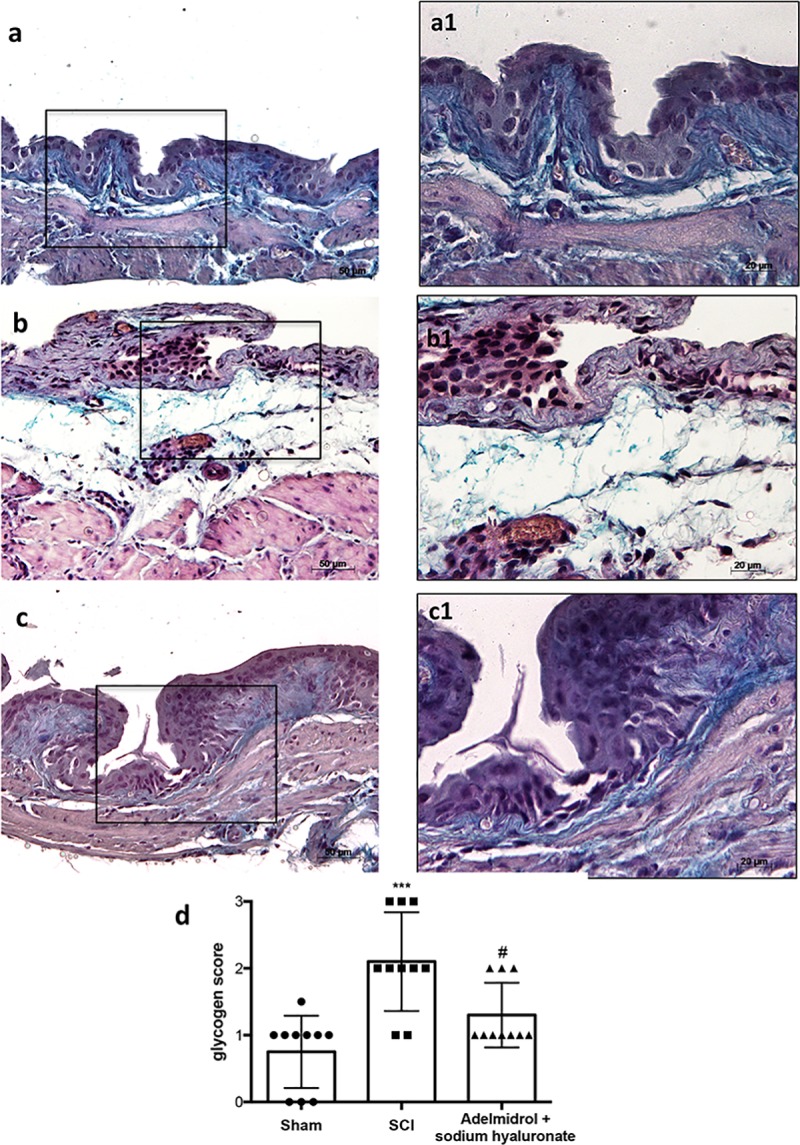
Chronic effects of adelmidrol + sodium hyaluronate on glycogen after SCI. PAS staining to identify the granular glycogen deposits in bladder tissue was performed at 7 days after SCI. Control mice showed no staining of glycogen granules (a, a1). Mice subjected to SCI presented an increase of positive staining of glycogen in the bladder (b, b1), while mice treated with adelmidrol + sodium hyaluronate showed a decreased of positive staining in the bladder (c, c1). These data are also presented graphically in the glycogen score (d) ***p< 0.001 *vs* Sham; ^#^p< 0.05 *vs* SCI.

PAS staining (Fig 7; S5 Table) showed an evident increase in goblet cell-producing mucus in the bladder 7 days after SCI (Fig 7B; higher magnification in 7B1 and relative histological analysis in 7D), compared to sham mice (Fig 7A; higher magnification in 7A1 and relative histological analysis in 7D). Daily treatment with adelmidrol + sodium hyaluronate for 7 days significantly decreased mucus production (Fig 7C; higher magnification in 7C1 and relative histological analysis 7D).

### Chronic adelmidrol + sodium hyaluronate effect on bladder tight junctions and urinary NGF after SCI

**Fig 8:**
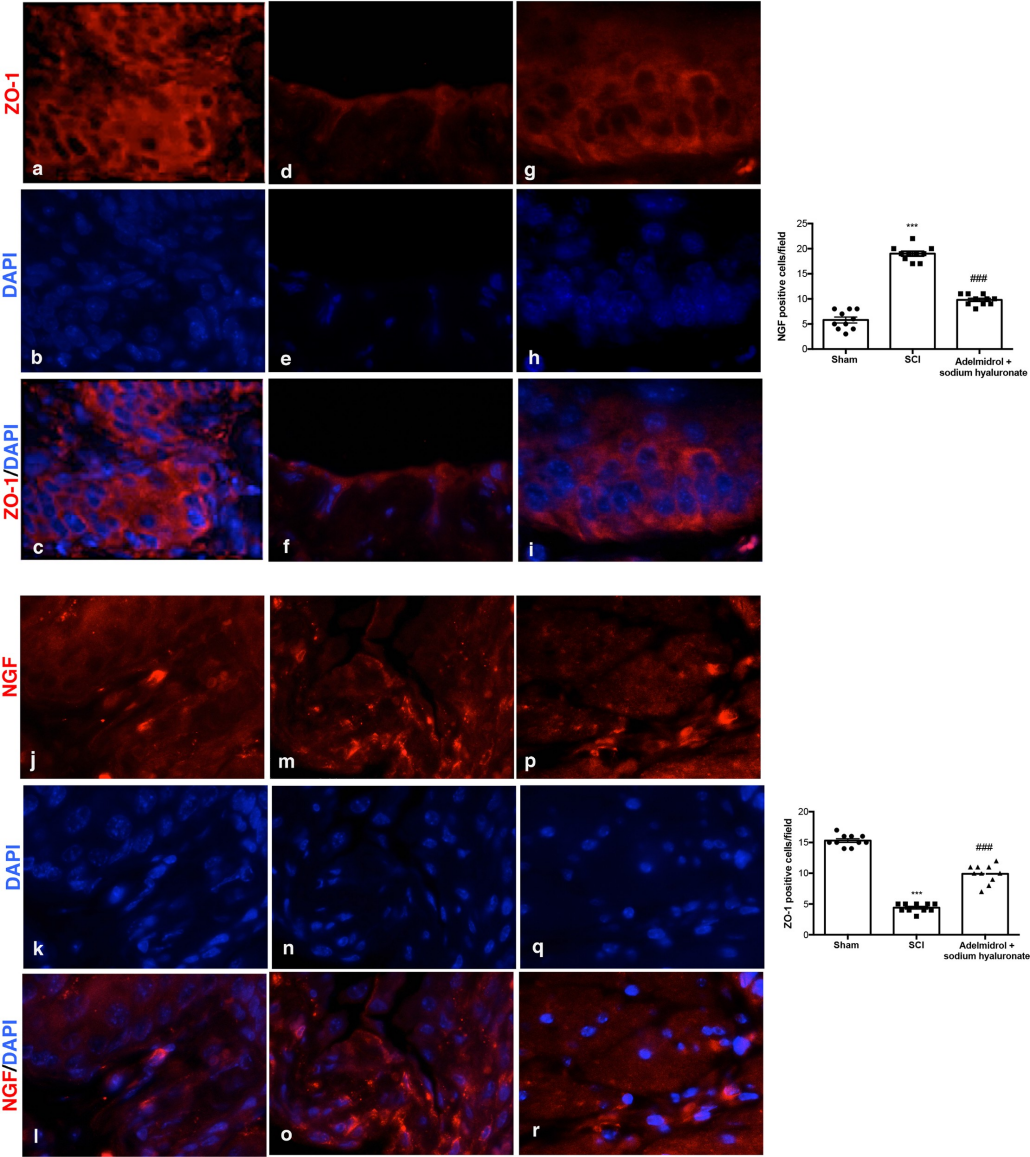
Chronic effects of adelmidrol + sodium hyaluronate on ZO-1 and NGF expression in bladder after SCI. Immunofluorescence for ZO-1 expression (red) in sham mice (a-c), SCI mice (d-f) and SCI mice treated with adelmidrol + sodium hyaluronate (g-i). Immunofluorescence for NGF expression (red) in sham mice (j-l), SCI mice (m-o) and SCI mice treated with adelmidrol + sodium hyaluronate (p-r). Data are representative of at minimum three independent experiments. Images are representative of all animals in each group. The graphs next to the respective panel represent the positive ZO-1 and NGF cells. All images were digitalized at a resolution of 8 bits into an array of 2048 x 2048 pixels. Pictures were captured at 100x magnification.

As demonstrated, acute inflammation leads to endothelial tight junction disassembly (ZO-1;Fig 8; S6 Table); accordingly, we observed, at 7 days, a decreased expression of ZO-1 in bladders of mice of SCI group (Fig 8D–8F) compared to control (Fig 8A–8C, see relative graph). Adelmidrol + sodium hyaluronate treatment increased significantly ZO-1 expression (Fig 8G–8I, see relative graph). Moreover, 7 days after SCI, mice showed a significantly increased NGF immunostaining (Fig 8M–8O, see relative graph), see relative graph, compared to sham animals (Fig 8J–8L, see relative graph). Adelmidrol + sodium hyaluronate treatment reduced markedly this NGF staining (Fig 8P–8R, see relative graph) (NGF;Fig 8; S7 Table).

### Chronic adelmidrol + sodium hyaluronate effect on proteinuria and urine bacterial growth

**Fig 9:**
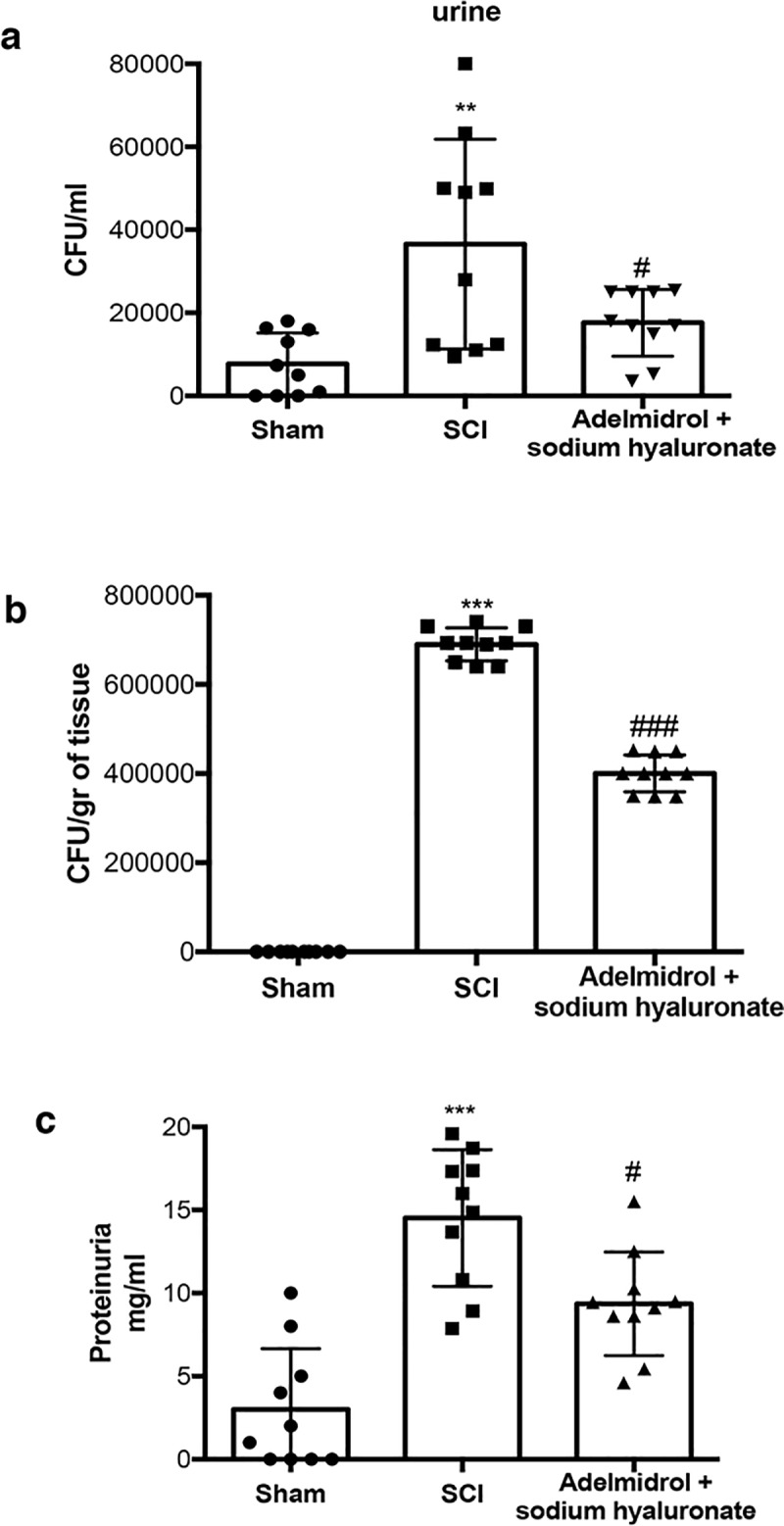
Chronic effects of adelmidrol + sodium hyaluronate on protein accumulation and bacterial growth in urine and in bladder of SCI mice. The Bradford assay showed an increase in protein in urine 7 days after SCI, adelmidrol + sodium hyaluronate treatment reduced significantly protein accumulation in urine (c). Mice subjected to SCI showed an increase of bacterial colonies in both urine and bladder, while treatment with adelmidrol + sodium hyaluronate, reduced significantly bacterial growth (a and b respectively). a) **p < 0.01 *vs* Sham; ^#^p < 0.05 *vs* SCI; b) ***p < 0.001 *vs* Sham; ^###^p < 0.001 *vs* SCI; c) ***p < 0.001 *vs* Sham; ^#^p < 0.05 *vs* SCI.

Urinary protein levels were assessed as an indicator of failure of the bladder barrier and/or the upper urinary tract (Fig 9; S8 Table). Protein in the urine and bladder was elevated within 7 days after SCI, compared to sham-injured mice. Interestingly, adelmidrol + sodium hyaluronate treatment significantly reduced protein accumulation in urine (Fig 9C) SCI patients can exhibit sustained ulceration of the bladder uroepithelium [28, 29]; this can leave the bladder susceptible to chronic cystitis or inflammation, possibly related to chronic bacterial infections. We thus examined the extent of bacterial growing in the urine and bladder 7 days after SCI. Mice subjected to SCI presented a marked increase in the number of bacterial colonies both in urine and bladder, which were significantly less in mice receiving treatment with adelmidrol + sodium hyaluronate (Fig 9A and 9B). All the relevant results of the study were collected in the table below (Table 1).

**Table 1:** Main study results in SCI mice treated with adelmidrol and sodium hyaluronate at 48h and 7d (Mean±SD).

	48h					7d			
GROUPS (Mice n = 10)	Glycogen score	MC	ZO-1	NGF	NF-κb	Glycogen score	ZO-1	NGF	Proteinuria
**Sham**	0.3±0.4	4.6±1.4	15.3±0.9	5.8±1.8	7842±5.1	0.75±0.5	18.3±2	2.2±1	3±3.6
**SCI**	2.1±0.7	50±6.6	4.4±0.7	19±1.5	19193±8	2.1±0.7	5.5±0.8	18.2±1.3	14.52±4.1
**SCI+ adelmidrol** **+ sodium hyaluronate**	1.3±0.5	25.4±4.3	9.9±1.5	9.8±1	9878±5	1.4±0.5	9.9±1.4	9.7±1.2	9.35±3.1

## Discussion

The consequences of SCI present a growing unmet medical need worldwide, especially among young adults who suffer a marked reduction quality of life [30]. Apart from the more clinically visible motor and sensory deficits, the majority of SCI patients experience also bladder dysfunction [31]. Loss of genitourinary function represents an important aspect in SCI. In fact, during SCI are interrupted the neuronal circuits that link elements of the peripheral and central nervous system, thereby jeopardizing bladder function [32]. Treatment of bladder dysfunction has improved in the last decades with a corresponding reduction in mortality. However, morbidity from bladder remains still a problem, as incontinence that can lead to recurrent infections associated with antibiotic resistance, kidney failure as well as social and professional isolation [33]. Therefore, improving bladder function is a fundamental priority among SCI patients [34, 35].

Adelmidrol, a semisynthetic derivative of azelaic acid, in the last years, has been considered as a successfully treatment for inflammatory disease [36]. It belongs to the ALIAmide family [37] showing anti-nociceptive and anti-inflammatory proprieties comparable to PEA in both *in vivo* and *in vitro* study [38, 39], as well as the capacity to modulate MCs hyper-reactivity in several pathophysiological circumstances [40, 41]. In addition, the combination of adelmidrol with hyaluronic acid improved the inflammatory signs of osteoarthritis induced by monosodium iodoacetate [19] as well as the protective effect of the combination with sodium hyaluronate on a model of cystitis demonstrating the anti-inflammatory properties in acute and chronic stage [17]. The current study was conducted to better understand the acute and chronic protective effects by intravesical administration of adelmidrol + sodium hyaluronate in an experimental model of SCI that induced bladder damage.

We first evaluated the acute effects of adelmidrol + sodium hyaluronate on bladder histological alteration after SCI. Bladder edema formation and epithelial ulceration was evident 48 h after SCI, which were reduced by intravesical adelmidrol + sodium hyaluronate treatment. Elevated glycogen content is a marker of irregular urodynamic function and an index of the severity of bladder functional changes after SCI. Bladder glycogen content during obstruction directly correlates with high pressures during voiding, lowered compliance and a high contractility. To evaluate this aspect of bladder after SCI we used PAS staining [24, 42]. This histochemical method showed positive staining of glycogen granules after intravesical treatment with adelmidrol + sodium hyaluronate. Data from clinical and animal models of acute and chronic urinary bladder inflammation indicate a central role for MCs [43]. An increased MC count is also characteristic of bladder alteration after SCI [44]. Here, bladder damage after SCI caused a important increase in MC numbers 48 h from damage; MC degranulation was significantly reduced by adelmidrol + sodium hyaluronate treatment. To confirm the effect of adelmidrol + sodium hyaluronate on activation and degranulation of MCs, we valued the expression of two proteases released by MCs, namely, chymase and tryptase [45]. A considerable increase in tryptase and chymase immunopositivity was evident in bladder collected 48 h after SCI, and this was reduced in SCI mice treated with adelmidrol + sodium hyaluronate.

Tight junctions in the urothelium, play a central role in development of the blood-urine barrier, and are important for epithelial normal function, paracellular circulation and conservation of cell surface polarity. Rickard et al [46] reported that the tight junction-associated proteins zonula occludens were concentrated at cell margins. Injuries to urothelial tight junctions interrupt barrier function, which induces urinary tract disorders [47]. In the present study, we found that the level of ZO-1 decreased in the bladder of mice of SCI group, while ZO-1 expression increased with adelmidrol + sodium hyaluronate treatment.

NF-κB represents a relevant factor in the production of pro-inflammatory mediators such as iNOS, involved in secondary inflammation associated with SCI [48]. The role of NF-κB in bladder inflammation is not well defined; however, in bladder biopsies from patients with interstitial cystitis, it has been observed an activation of NF-κB and its nuclear translocation[49]. Here, adelmidrol + sodium hyaluronate was able to inhibit the NF-κB pathway and decrease levels of iNOS in the bladder at 48 h after SCI.

The neurotrophin NGF plays a dynamic role in the bladder of patients with SCI, supposedly modulating neuronal cell function linked with micturition and also development of neurogenic bladder at the spinal level [50, 51]. Increased levels of NGF in the bladder accompanied the experimental models of SCI causing neurogenic or cyclophosphamide-induced cystitis [52]. In the present study, during the acute condition an increase in NGF immunoreactivity was found in the bladder of SCI mice, which was significantly diminished in bladder tissue collected from SCI mice treated with adelmidrol + sodium hyaluronate.

In a second set of studies we evaluated the effect of treatment with adelmidrol + sodium hyaluronate at longer times. At 7 days following injury, SCI mice untreated displayed histological bladder alterations, neutrophil infiltration, a marked increasing of goblet cell-producing mucus and an increase in protein and bacterial colonies in the urine and bladder, all of which were significantly reduced by adelmidrol + sodium hyaluronate treatment. Further, bladder immunoreactivity of ZO-1 was decreased in SCI mice, but treatment with adelmidrol + sodium hyaluronate maintained high levels of ZO-1 even 7 days post-injury. NGF levels are notably higher in inflamed tissues, and may contribute to chronic inflammation and damage [53]. NGF immunoreactivity was also elevated in bladder tissue of 7-day SCI mice, with adelmidrol + sodium hyaluronate treatment significantly reducing the rise in NGF levels.

In conclusion we demonstrate, for the first time, that intravesical administration of adelmidrol + sodium hyaluronate possesses an anti-inflammatory effect in acute and chronic models of SCI that cause bladder damage. Thus, adelmidrol + sodium hyaluronate could represent a new research focus in the quest for better treatments for bladder dysfunction associated with SCI. We suggest that adelmidrol + sodium hyaluronate action may be due, in part, to modulation of MC activation, to inhibition of inflammatory pathways such as NF-κB, as well as in the reduction of bladder dysfunction by the modulation of NGF. However, further studies are needed to better understand the adelmidrol + sodium hyaluronate mechanism.

### Availability of data and material

The authors declare that all data supporting the findings of this study are available within the article. The data that support the findings of this study are available from the corresponding author upon reasonable request.

## Supporting information

S1 Fig Original blots of Fig 5. (PDF)

S1 Table Raw data of glicogen score at 48h. (DOCX)

S2 Table Raw data of mast cells (trypan blue assay). (DOCX)

S3 Table Raw data of immunofluorescence staining ZO-1/DAPI at 48h. (DOCX)

S4 Table Raw data of immunofluorescence staining NGF/DAPI at 48h. (DOCX)

S5 Table Raw data of glicogen score at 7d. (DOCX)

S6 Table Raw data of immunofluorescence staining ZO-1/DAPI at 7d. (DOCX)

S7 Table Raw data of immunofluorescence staining NGF/DAPI at 7d. (DOCX)

S8 Table Raw data of proteinuria assay at 7d. (DOCX)
