# Supplementary material for: The association of adelmidrol with sodium hyaluronate displays beneficial properties against bladder changes following spinal cord injury in mice
Source: PLoS One. 2019 Jan 17;14(1):e0208730. doi: 10.1371/journal.pone.0208730 (PMC6336272; doi:10.1371/journal.pone.0208730)
Supplement: S1 Fig — (PDF) [file pone.0208730.s001.pdf]

The association of adelmidrol with sodium hyaluronate displays beneficial properties against bladder changes following spinal cord injury in mice.

Short title: Effect of adelmidrol and sodium hyaluronate on bladder after spinal cord injury

Michela Campolo[a#], Rosalba Siracusa[a #], Marika Cordaro[a], Alessia Filippone[a], Enrico Gugliandolo[a], Alessio F. Peritore[a], Daniela Impellizzeri[a], Rosalia Crupi[a], Irene Paterniti[a], Salvatore Cuzzocrea[a,b*].

[a] University of Messina, Department of Chemical, Biological, Pharmaceutical and Environmental Sciences, Messina, Italy

[b] Saint Louis University School of Medicine, Department of Pharmacological and Physiological Science, Saint Louis, USA

[#]These authors contributed equally to this work

***Corresponding Author: Prof. Salvatore Cuzzocrea**, Department of Chemical, Biological, Pharmaceutical and Environmental Sciences, University of Messina, Viale Ferdinando Stagno D'Alcontres n°31 98166 Messina, Italy; Tel.: +39 090-6765208, email: salvator@unime.it

Full images of Western bots for Figure 5

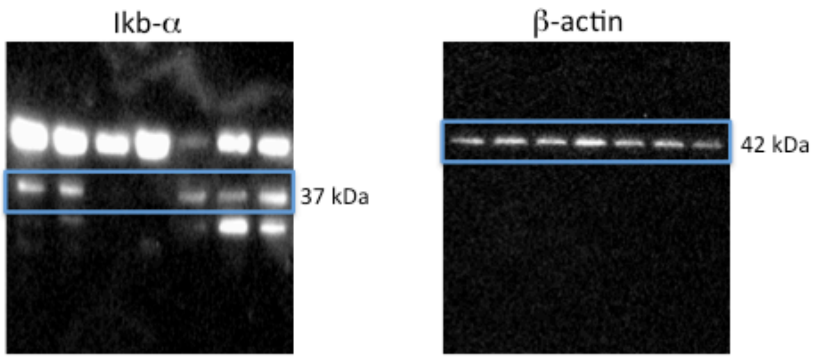

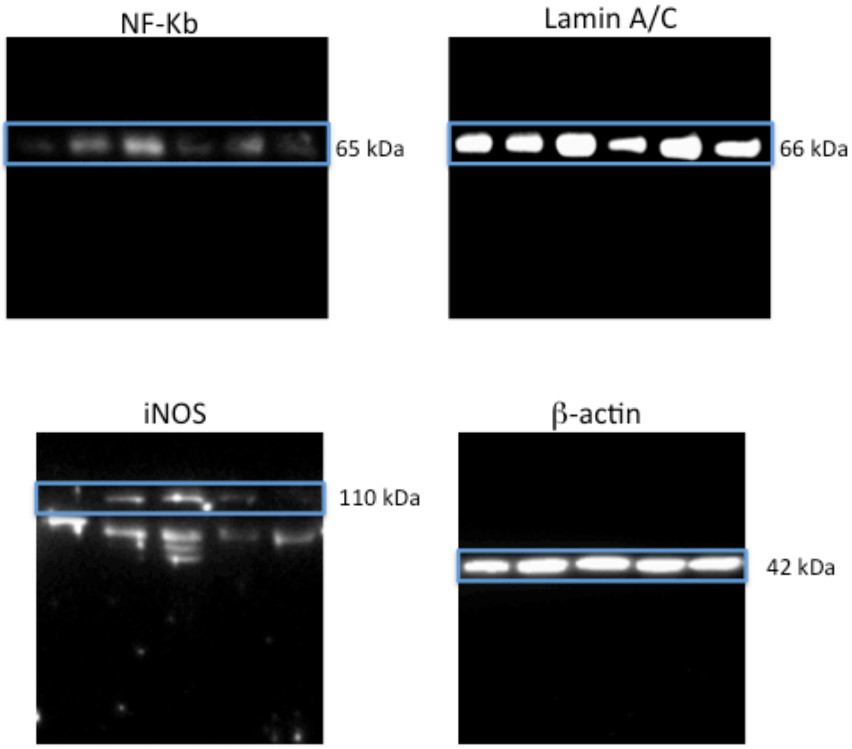

Suppl.Fig 5. original blot of Figure 5
