# Supplementary material for: The association of adelmidrol with sodium hyaluronate displays beneficial properties against bladder changes following spinal cord injury in mice
Source: PLoS One. 2019 Jan 17;14(1):e0208730. doi: 10.1371/journal.pone.0208730 (PMC6336272; doi:10.1371/journal.pone.0208730)
Supplement: S1 Table — (DOCX) [file pone.0208730.s002.docx]

**Table 1. Glicogen score 48h**

**Mice n=10**

| **Sham** | **SCI** | **SCI+ 2% adelmidrol + 0.1% sodium hyaluronate** |
| --- | --- | --- |
| 1 | 2 | 1 |
| 0 | 2 | 1 |
| 0 | 2 | 1 |
| 0 | 3 | 2 |
| 0 | 1 | 2 |
| 1 | 2 | 1 |
| 0 | 3 | 2 |
| 1 | 2 | 1 |
| 0 | 3 | 2 |
| 0 | 1 | 1 |

| **Mean** | 0,3 | 2,1 | 1,4 |
| --- | --- | --- | --- |
| **Std. Deviation** | 0,483 | 0,7379 | 0,5164 |
| **Std. Error of Mean** | 0,1528 | 0,2333 | 0,1633 |
