# Supplementary material for: The association of adelmidrol with sodium hyaluronate displays beneficial properties against bladder changes following spinal cord injury in mice
Source: PLoS One. 2019 Jan 17;14(1):e0208730. doi: 10.1371/journal.pone.0208730 (PMC6336272; doi:10.1371/journal.pone.0208730)
Supplement: S2 Table — (DOCX) [file pone.0208730.s003.docx]

**Table 2. Mast cells (trypan blue assay)**

**Mice n=10**

| **Sham** | **SCI** | **SCI+ 2% adelmidrol+ 0,1%sodium hyaluronate** |
| --- | --- | --- |
| 5 | 50 | 30 |
| 6 | 45 | 25 |
| 2 | 60 | 30 |
| 5 | 50 | 20 |
| 3 | 50 | 22 |
| 5 | 45 | 30 |
| 6 | 55 | 25 |
| 3 | 60 | 30 |
| 5 | 45 | 20 |
| 6 | 40 | 22 |

| **Mean** | 4,6 | 50 | 25,4 |
| --- | --- | --- | --- |
| **Std. Deviation** | 1,43 | 6,667 | 4,3 |
| **Std. Error of Mean** | 0,4522 | 2,108 | 1,36 |
