# Supplementary material for: The association of adelmidrol with sodium hyaluronate displays beneficial properties against bladder changes following spinal cord injury in mice
Source: PLoS One. 2019 Jan 17;14(1):e0208730. doi: 10.1371/journal.pone.0208730 (PMC6336272; doi:10.1371/journal.pone.0208730)
Supplement: S3 Table — (DOCX) [file pone.0208730.s004.docx]

**Table 3. Immunofluorescence staining ZO-1/DAPI 48h**

**Mice n=10**

| **Sham** | **SCI** | **SCI+ 2% adelmidrol+ 0,1%sodium hyaluronate** |
| --- | --- | --- |
| 15 | 5 | 10 |
| 16 | 4 | 12 |
| 14 | 5 | 11 |
| 15 | 3 | 11 |
| 16 | 5 | 7 |
| 15 | 4 | 11 |
| 14 | 4 | 10 |
| 16 | 5 | 8 |
| 17 | 4 | 9 |
| 15 | 5 | 10 |

| **Mean** | 15,3 | 4,4 | 9,9 |
| --- | --- | --- | --- |
| **Std. Deviation** | 0,9487 | 0,6992 | 1,524 |
| **Std. Error of Mean** | 0,3 | 0,2211 | 0,4819 |
