# Supplementary material for: The association of adelmidrol with sodium hyaluronate displays beneficial properties against bladder changes following spinal cord injury in mice
Source: PLoS One. 2019 Jan 17;14(1):e0208730. doi: 10.1371/journal.pone.0208730 (PMC6336272; doi:10.1371/journal.pone.0208730)
Supplement: S4 Table — (DOCX) [file pone.0208730.s005.docx]

**Table 4. Immunofluorescence staining NGF/DAPI 48h**

**Mice n=10**

| **Sham** | **SCI** | **SCI+ 2% adelmidrol+ 0,1%sodium hyaluronate** |
| --- | --- | --- |
| 8 | 19 | 9 |
| 3 | 17 | 10 |
| 5 | 22 | 10 |
| 4 | 19 | 11 |
| 7 | 17 | 11 |
| 8 | 20 | 9 |
| 8 | 19 | 9 |
| 5 | 20 | 8 |
| 4 | 19 | 10 |
| 6 | 18 | 11 |

| **Mean** | 5,8 | 19 | 9,8 |
| --- | --- | --- | --- |
| **Std. Deviation** | 1,874 | 1,491 | 1,033 |
| **Std. Error of Mean** | 0,5925 | 0,4714 | 0,3266 |
