# Supplementary material for: The association of adelmidrol with sodium hyaluronate displays beneficial properties against bladder changes following spinal cord injury in mice
Source: PLoS One. 2019 Jan 17;14(1):e0208730. doi: 10.1371/journal.pone.0208730 (PMC6336272; doi:10.1371/journal.pone.0208730)
Supplement: S6 Table — (DOCX) [file pone.0208730.s007.docx]

**Table 6. Immunofluorescence staining ZO-1/DAPI 7g**

**Mice n=10**

| **Sham** | **SCI** | **SCI+ 2% adelmidrol+ 0,1%sodium hyaluronate** |
| --- | --- | --- |
| 18 | 5 | 10 |
| 16 | 6 | 12 |
| 20 | 5 | 9 |
| 15 | 6 | 11 |
| 17 | 5 | 8 |
| 22 | 6 | 10 |
| 18 | 6 | 10 |
| 18 | 7 | 12 |
| 19 | 4 | 8 |
| 20 | 5 | 9 |

| **Mean** | 18,3 | 5,5 | 9,9 |
| --- | --- | --- | --- |
| **Std. Deviation** | 2,058 | 0,8498 | 1,449 |
| **Std. Error of Mean** | 0,6506 | 0,2687 | 0,4583 |
