# Supplementary material for: The association of adelmidrol with sodium hyaluronate displays beneficial properties against bladder changes following spinal cord injury in mice
Source: PLoS One. 2019 Jan 17;14(1):e0208730. doi: 10.1371/journal.pone.0208730 (PMC6336272; doi:10.1371/journal.pone.0208730)
Supplement: S8 Table — (DOCX) [file pone.0208730.s009.docx]

**Table 8. Proteinuria assay 7d**

**Mice n=10**

| **Sham** | **SCI** | **SCI+ 2% adelmidrol+ 0,1%sodium hyaluronate** |
| --- | --- | --- |
| 0 | 8,924427 | 9,490969 |
| 4 | 7,880177 | 10,25547 |
| 0 | 10,81315 | 8,613374 |
| 0 | 19,590969 | 9,435443 |
| 1 | 17,32287 | 12,51 |
| 2 | 18,724463 | 4,613374 |
| 0 | 14,87776 | 5,435443 |
| 5 | 13,6791 | 15,51 |
| 8 | 15,99876 | 9,110969 |
| 10 | 17,37887 | 8,613374 |

| **Mean** | 3 | 14,52 | 9,359 |
| --- | --- | --- | --- |
| **Std. Deviation** | 3,651 | 4,104 | 3,121 |
| **Std. Error of Mean** | 1,155 | 1,298 | 0,987 |
